# An Antimicrobial and Antifibrotic Coating for Implantable Biosensors

**DOI:** 10.3390/bios15030171

**Published:** 2025-03-06

**Authors:** Sofia Wareham-Mathiassen, Pawan Jolly, Nandhinee Radha Shanmugam, Badrinath Jagannath, Pranav Prabhala, Yunhao Zhai, Alican Ozkan, Arash Naziripour, Rohini Singh, Henrik Bengtsson, Thomas Bjarnsholt, Donald E. Ingber

**Affiliations:** 1 Wyss Institute for Biologically Inspired Engineering, Harvard University, Boston, MA 02215, USA; sfwm@novonordisk.com (S.W.-M.); pawan.jolly@wyss.harvard.edu (P.J.); nandhinee.radhashanmugam@wyss.harvard.edu (N.R.S.); badrinath.jagannath@wyss.harvard.edu (B.J.); pranav.prabhala@wyss.harvard.edu (P.P.); yunhao.zhai@wyss.harvard.edu (Y.Z.); alican.ozkan@wyss.harvard.edu (A.O.); arash.naziripour@wyss.harvard.edu (A.N.); rohini.singh@wyss.harvard.edu (R.S.); 2 Novo Nordisk A/S, 2880 Bagsværd, Denmark; hbss@novonordisk.com; 3 Costerton Biofilm Center, Copenhagen University, 2200 Copenhagen, Denmark; tbjarnsholt@sund.ku.dk; 4 Harvard John A. Paulson School of Engineering and Applied Sciences, Cambridge, MA 02134, USA; 5 Vascular Biology Program and Department of Surgery, Boston’s Children’s Hospital and Harvard Medical School, Boston, MA 02115, USA

**Keywords:** antifouling, antimicrobial, diagnostics, electrochemical sensor, multiplexing, wearable, implant, glucometer

## Abstract

Biofouling and foreign body responses have deleterious effects on the functionality and longevity of implantable biosensors, seriously impeding their implementation for long-term monitoring. Here, we describe a nanocomposite coating composed of a cross-linked lattice of bovine serum albumin and pentaamine-functionalized reduced graphene that is covalently coupled to antibody ligands for analyte detection as well as antibiotic drugs (gentamicin or ceftriaxone), which actively combats biofouling while retaining high electroconductivity and excellent electrochemical immunosensor behavior. Sensors overlaid with this coating inhibit the proliferation of *Pseudomonas aeruginosa* bacteria and adhesion of primary human fibroblasts while not having any significant effects on fibroblast viability or on the immune function of primary human monocytes. Under these conditions, the sensor maintains its electrochemical stability for at least 3 weeks after exposure to soluble proteins that interfere with the activity of uncoated sensors. Proof-of-concept for the coating’s applicability is demonstrated by integrating the antimicrobial coating within an immunosensor and demonstrating the detection of cytokines in both culture medium and complex human plasma. This new coating technology holds the potential to substantially increase the lifespan of implanted biosensors and widen their application areas, potentially enabling continuous monitoring of analytes in complex biofluids for weeks in vivo.

## 1. Introduction

The implementation of wearable electrochemical biosensors, such as implantable glucometers used for the management of patients with diabetes, is greatly hindered by biofouling as non-specific absorption of proteins and adhesion of host cells passivate and thereby deteriorate the electrode, compromising electrochemical performance [1,2]. Implantation of a foreign body also elicits unfavorable tissue reactions known as the foreign body response (FBR), physically isolating the sensor from analytes of interest, compromising device functionality, and fostering subsequent biofilm formation, all of which contribute to an elevated risk of infection and implant failure [3,4,5,6,7]. Thus, mitigating biofouling, improving biocompatibility, and minimizing both acute and chronic FBRs are crucial for the successful clinical translation of biosensors in medical applications that require continuous sensing of analytes for extended periods of time (weeks or longer) in vivo.

To provide coatings with active antimicrobial features, common strategies involve embedding or releasing microbicidal agents, such as ions, nanoparticles, salts, antibiotics, or other antimicrobial molecules [8,9,10]. While highly effective in the short term, coatings that release soluble antimicrobial agents encounter challenges regarding loading capacity, reservoir exhaustion, cellular toxicity, and potential induction of antimicrobial resistance, which restricts their long-term use [11]. While coatings employing contact-killing methods can be effective without exerting adverse effects on surrounding tissue, they are prone to biofouling as dead bacteria accumulate on their surfaces [11,12,13].

While efforts to mitigate biofouling of sensor surfaces are widespread, few studies have successfully incorporated antimicrobial agents [14,15,16]. Silver and gold nanoparticle coatings have been investigated for their antimicrobial properties, but they often fall short of providing the electrochemical performance required for sensitive and selective analyte detection. Silver coatings, in particular, are prone to oxidation in air and can undergo electrochemical oxidation during readout, leading to background current interference that compromises sensor accuracy [17,18,19]. This challenge is further compounded by a trade-off between coating thickness and functionality: thin films (5–15 nm) optimize electrochemical performance, whereas thicker antimicrobial coatings, such as zinc oxide (200–600 nm) and silver nanoparticles (20–300 nm), compromise sensor sensitivity [19,20,21,22,23]. Efficiently incorporating an antimicrobial agent into thinner coatings also poses challenges, as seen in past studies on gold nanoparticle-loaded nanocomposites that revealed issues with integration and bovine serum albumin (BSA) adsorption, which appear to be due to electrostatic repulsion [24]. While recent studies have successfully integrated antimicrobial properties into wearable, flexible sensor coatings for sweat analyte detection, biocompatibility and antifibrotic capabilities for indwelling or implantable use have yet to be demonstrated [25,26].

In response to these challenges, we set out to develop a safe, thin film nanocomposite coating that would inhibit microbial adhesion and growth while maintaining excellent electrochemical properties for implantable or wearable electrochemical sensor applications. This is the first of its kind. We previously reported a highly effective antifouling coating for electrochemical sensors that are used outside the body (e.g., for point-of-care diagnostic applications). This antifouling coating is a three-dimensional (3D) nanocomposite composed of electroconductive pentaamine-functionalized, reduced graphene oxide flakes (prGOx) embedded within a BSA gel that is stabilized by crosslinking with glutaraldehyde (GTA), resulting in a BSA/prGOx/GTA nanocomposite [15,24]. This unique structure facilitates electron transfer while effectively preventing protein diffusion, serving as a protective shield for the electrode [24,27]. However, there are potential cytotoxicity issues associated with the use of GTA due to the presence of unreacted moieties and toxic by-products, which limit its applicability for coating electrochemical sensors in wearable or implantable devices [28,29].

Here, we modified the previously published BSA/prGOx/GTA coating by replacing GTA with the biocompatible crosslinker, genipin (GNP), and instilled it with antimicrobial activity by incorporating crosslinked antibiotics (ab) into the matrix, resulting in a coating technology (collectively referred to as BSA/prGOx/GNP/ab) that exhibits antimicrobial activity through active and passive strategies while retaining outstanding electrochemical behavior over weeks. The BSA/prGOx/GNP/ab covalently linked antibiotics confer the coating with a sustained, non-leaching antimicrobial effect, preventing leaking and eventual exhaustion of the antimicrobial particles, while maintaining a thickness appropriate for electrochemical conductance. This coating also enhances biocompatibility by preventing the attachment and activation of host stromal and immune cells, which can contribute to localized discomfort, irritation, pain, erythema, and edema, as well as the initiation of an FBR [5,30,31]. Proof of concept is demonstrated by integrating this coating into an antibody-based electrochemical sensor for two important inflammatory biomarkers, MIP-1β and IL-6, and demonstrating its functionality in complex human plasma for at least 3 weeks in vitro. This antimicrobial coating technology offers high cost-effectiveness and manufacturability due to a single-pot recipe of low-cost components and the use of a highly scalable drop-casting method. It also has the potential to substantially increase the lifespan and functionality of implantable biosensors, presenting exciting avenues for continuous in vivo monitoring of a plethora of analytes.

## 2. Materials and Methods

### 2.1. Coating Synthesis

#### 2.1.1. Antifouling Nanocomposite Preparation

The BSA/prGOx/GTA nanocomposite was prepared and drop-cast on custom-fabricated gold electrode chips (Telic Company, Santa Clarita, CA, USA) as previously described and further detailed in the Supplementary Materials [15,27]. The antimicrobial, biocompatible nanocomposite BSA-GNP was similarly prepared by sonicating 8 mg mL^−1^ of prGOx nanoflakes (Millipore Sigma, Burlington, MA, USA, no. 806 579) with 5 mg mL^−1^ BSA (IgG-Free, Protease-Free; Jackson ImmunoResearch, West Grove, PA, USA, no. 001-000-162) in a 10 × 10^−3^ m phosphate-buffered saline solution (PBS, pH 7.4) (Sigma-Aldrich, Burlington, MA, USA, no. D8537). The solution mixture was sonicated by a tip sonicator for 30 min using 1 s on/off cycles at 50% amplitude, 125 W, and 20 kHz (Bransonic, Danbury, CT, USA, CPX 3800), followed by heating (Labnet, Edison, NJ, USA, no. D1200) at 105 °C for 5 min to denature the protein. The resulting opaque black mixture was centrifuged at 16.2 RCF for 15 min to remove excess aggregates. The semitransparent nanocomposite supernatant solution was then mixed either with 70% glutaraldehyde (Sigma-Aldrich, Burlington, MA, USA, no. G7776) for BSA/prGOx/GTA or GNP (Adooq Bioscience, Irvine, CA, USA, no. A11677) dissolved in 50% ethanol at 1 mg mL^−1^ for BSA/prGOx/GNP at a ratio of 69:1.

#### 2.1.2. Addition of Cross-Linked Antibiotics

As a proof of concept, we first crosslinked antibiotics with primary amine groups to the BSA/prGOx nanocomposite to actively mitigate microbial adhesion and prevent the leaching of antibiotics. Specifically, we made various BSA/prGOx/GNP using different concentrations of each antibiotic. Briefly, gentamicin sulfate salt (G), cefepime (C), ceftriaxone (Cx), and colistin (Co) (all from Sigma-Aldrich, Burlington, MA, USA) were dissolved in deionized (DI) water at 70 mg mL^−1^ and added to the prGOx/BSA/GNP coating, 1 µL per chip for a final concentration of 1 mg mL^−1^, or diluted further to 0.1 mg mL^−1^.

#### 2.1.3. Coating of Antifouling Nanocomposite onto Electrodes

For each antifouling nanocomposite, 70 μL was drop-cast over the surface of gold electrodes that were pre-cleaned and plasma-treated as previously described [24], incubated in a humidity chamber overnight at room temperature, then rinsed and washed with PBS at 400 rpm for 2 min. Double-sided adhesive tape was used to create a well for liquids (ARseal90 880 Polypropylene Double-Sided Adhesive Tape, Adhesive Research Inc., Glen Rock, PA, USA). Chips were then exposed to 1 M ethanolamine (Sigma-Aldrich, Burlington, MA, USA, no. E9508) in PBS to quench the unreacted GTA and GNP groups before further immobilization steps.

#### 2.1.4. Fabrication of Electrochemical Immunosensor

Chips with coated electrodes were exposed to a solution containing the GNP-G crosslinked prGOx/BSA nanocomposite overnight as described in the aforementioned section to fabricate an electrochemical immunosensor. Chips were then washed in PBS at 400 rpm for 10 min and dried. Coated chips were functionalized with 400 mM EDC (Thermo Fischer Scientific, Waltham, MA, USA) and 200 mM NHS (Sigma-Aldrich, Burlington, MA, USA) dissolved in 0.05 M MES for 30 min, rinsed with DI water, and dried at RT. Three working electrodes were spotted with the optimized concentration of respective capture antibodies (anti-MIP (R&D systems) and anti-IL-6 (Abcam, Cambridge, UK)) diluted in PBS, and the fourth electrode was spotted with a negative control (BSA) using an Xtend capillary microarray pin (LabNEXT), and all electrodes were incubated in a humidity chamber at 4 °C overnight. Chips were washed with PBS and treated with 1 M ethanolamine for 30 min to quench the unreacted GNP groups, followed by blocking with 2.5% BSA in PBS for 1 h. MIP antigen (R&D systems) was spiked into RPMI (RPMI 1640, Gibco, 72400-047) ranging from 0.01 to 100 ng mL^−1^ and chips were incubated with 15 μL of (MIP antigen + RPMI) samples for 1 h. Similarly, IL-6 antigen (Abcam, Cambridge, UK) was spiked into RPMI or human plasma (Innovative Research, USA no. IPLAS-COV2P100UL), and chips were incubated with 15 µL of (IL-6 antigen + RPMI or human plasma) samples for 1 h. Chips were rinsed with 0.05% PBST and treated with their respective biotinylated detection antibodies. Biotinylation of the MIP detection antibody was achieved using a Biotin Conjugation Kit (Abcam, Cambridge, UK) and the IL-6 from the ab246838 Human IL-6 Matched Antibody Pair Kit (Abcam, Cambridge, UK). The MIP and IL-6 sensors were incubated with MIP DAb and IL-6 DAb for 15 and 30 min, respectively, and then washed with 0.05% PBST. DAb-treated sensors were exposed to 5 mg mL^−1^ of Poly-HRP-Streptavidin (Thermo Fisher Scientific) prepared in 0.1% BSA in PBST for 5 min and then rinsed with PBST. Precipitating TMB (Sigma-Aldrich, Burlington, MA, USA) was added to the MIP sensor for 1 min and to the IL-6 sensor for 3 min. Finally, sensors were rinsed with PBST, and electrochemical measurements were taken.

### 2.2. Electrochemical Characterization of Antifouling Nanocomposite

Electrochemical characterizations were carried out as described previously [15]. Briefly, each chip contained four gold electrodes, a working electrode, and a reference electrode connected to the potentiostat (Autolab PGSTAT128N, Metrohm; VSP, Bio-Logic, Seyssinet-Pariset, France) through a connector built in-house. Electrochemical characterization of the antifouling nanocomposite was characterized by cyclic voltammograms (CV) in a redox aqueous solution of PBS containing 5 × 10^−3^ M potassium ferro/ferricyanide [Fe(CN)6]^3−/4−^ at a scan rate of 1 V/s between −0.5 and 0.5 V. The antifouling capacity of the nanocomposite coating was tested after incubation with 1% BSA for 1 h and 24 h. The immunosensors were then characterized using CV in PBST at a scan rate of 1 V s^−1^ between −0.5 and 0.5 V. The on-chip integrated (uncoated) gold electrode was used as a quasi-reference electrode for all electrochemical measurements. The LoD is determined using the formula LOD = 3.3 * (**σ**_blank_ m^−1^), where **σ**_blank_ is the standard deviation of the blank and m is the slope of the calibration curve.

### 2.3. Antimicrobial Efficacy Evaluation

#### 2.3.1. Coating of Microtiter Plate

The antimicrobial efficacy of each nanocomposite coating was evaluated using a broth dilution assay. Briefly, a Falcon™ 96-Well, Cell Culture-Treated, Flat-Bottom Microplate (Thermo Fisher Scientific, Waltham, MA, USA) was coated with the respective combinations of nanocomposites in triplicate (43 µL per well) by the drop-casting method as described above, followed by incubation in a humidity chamber overnight at room temperature and washing with PBS. Coatings were then quenched by adding 1 M ethanolamine (6 µL ethanolamine to 94 µL PBS) for 30 min at room temperature.

#### 2.3.2. Inoculation with Bacteria

*Pseudomonas aeruginosa* (*P. aeruginosa*) ATCC 10145 was cultured on LB agar plates. Fresh colonies were picked and placed in 5 mL LB medium at 37 °C for 8 h, which was then diluted at 1:500 to 1:1000 to inoculate the overnight culture. The overnight culture was then adjusted with the BD PhoenixSpec™ nephelometer to 0.5 ± 0.05 MacFarland and diluted to 1:20 in LB medium. To each treatment and control well, 100 µL LB medium was added, followed by 100 µL of the diluted bacterial suspension for a final concentration of ~2 × 10^6^ CFU mL^−1^ per well, verified by plating. Absorbance was measured every 30 min for 10–15 h under shaking at OD 600 and 37 °C using the Synergy H1 Hybrid Multi-Mode Reader (Agilent technologies, Santa Clara, CA, USA) with BioTek Gen5 software.

### 2.4. Biocompatibility Evaluation

#### 2.4.1. Coating of Wells

Nanocomposite coatings of different combinations were tested: BSA/prGOx/GTA, BSA/prGOx/GNP, BSA/prGOx/GNP/G, and BSA/prGOx/GNP/Cx. A polyurethane (PU) film containing 0.1% zinc diethyldithiocarbamate (ZDEC) (Hetano Research Institute, Food and Drug Safety Center, Hadano, Japan) was used as a positive control and an uncoated tissue culture plate as a negative control [32]. Falcon™ 96-Well, Cell Culture-Treated, Flat-Bottom Microplates (Thermo Fisher Scientific, Waltham, MA, USA) were coated using the drop-casting method described above with each nanocomposite combination.

#### 2.4.2. Monocyte Isolation and Culture

De-identified human patient-derived apheresis collars (a by-product of platelet isolation) were obtained from the Crimson Biomaterials Collection Core Facility under approval from the Institutional Review Board at Harvard University (#22470); informed written consent was not required. PBMCs were isolated by density centrifugation using Lymphoprep (StemCell Technologies, Vancouver, BC, Cananda, 07801), and magnetic beads were used for negative selection of monocytes (StemCell Technologies, Vancouver, BC, Cananda, 19058). Monocytes were then seeded at approximately 100,000 cells per well and cultured under the various experimental conditions in complete RPMI, 10% fetal bovine serum (FBS, Thermo Fisher Scientific, Waltham, MA, USA, 10082-147), and 1% Penicillin/Streptomycin (Thermo Fisher Scientific, Waltham, MA, USA, 15140122). Supernatants from each condition and timepoint were then collected and stored at −20 °C for MSD analysis; live cells were harvested and analyzed by flow cytometry, the alamarBlue™ Cell Viability assay, MDS analysis, and microscopy.

#### 2.4.3. Fibroblast Isolation and Culture

Full-thickness samples were isolated anonymously from healthy regions of human colon resections and processed in the Department of Pathology at Massachusetts General Hospital under an existing Institutional Review Board (approved protocol number is #2015P001859). Deidentified samples (macroscopically grossly unaffected regions of the colon) were collected from adult patients undergoing endoscopy for abnormal complaints. To isolate fibroblasts from the stromal tissue, all collected samples were pooled and digested with 2 mg mL^−1^ collagenase I (17100-017; Thermo Fisher Scientific, Waltham, MA, USA) supplemented with 10 mmol L^−1^ Y-27632 (Y0503; Sigma-Aldrich, Burlington, MA, USA) for 40 min at 37 °C with intermittent agitation, then suspended in fibroblast growth medium (FGM-2, Lonza, Basel, Switzerland, CC-3131) supplemented with a bullet kit (Lonza, Cat # CC-4126) for further expansion. Culture medium was changed every other day to provide enough nutrients for proliferation. Cells were seeded on the nanocomposite-coated 96-well plates, allowed to reach 70% confluency, and incubated for 24 and 48 h at 37 °C. Supernatant from each condition was then collected.

#### 2.4.4. Flow Cytometry

Cells for flow cytometry analysis were harvested via pipetting, centrifuged at 300 rfc for 5 min, and resuspended in PBS for staining. Cells were first labeled with ViaKrome fixable viability dye (1:150 dilution; Beckman Coulter, Brea, CA, USA, C36628), followed by a 30 min incubation with fluorophore-labeled antibodies and Fc Block (1:100 dilution) at 4 °C. Cells were washed twice with PBS and then fixed with Cytofix (BD Biosciences, Franklin Lakes, NJ, USA, 554655) for 15 min at 4 °C. Cells were centrifuged and re-suspended in PBS containing 2% FBS and 2 mM EDTA and stored at 4 °C prior to analysis using a Cytoflex LX flow cytometer (Beckman Coulter, Brea, CA, USA). Results were analyzed using FlowJo V10 software (FlowJo, Ashland, OR, USA, LLC). Antibodies to CD80 (#305232), CD40 (#334345), HLA-DR (#361610), CD1c (#331526), and CD86 (# 305430) were obtained from Biolegend (Biolegend, San Diego, CA, USA); antibodies to CD83 (#19677) were obtained from Santa Cruz Biotechnology; antibodies to CD14 (#563561) were obtained from BD (BD Biosciences, Franklin Lakes, NJ, USA).

#### 2.4.5. Cell Viability Assay

To assess cell metabolic activity, monocytes and fibroblasts were treated with alamarBlue™ Cell Viability Reagent (Thermo Fisher Scientific, Waltham, MA, USA). Cells were washed and resuspended in fresh RPMI with alamarBlue™ Cell Viability Reagent at 10% total volume and incubated at 37 °C. Fluorescence was measured using Biotek fluorescence spectrophotometry (Biotek, Winooski, VT, USA) at an excitation wavelength of 560 nm and an emission wavelength of 590 nm. Measurements were taken every 10 min for 1 h.

#### 2.4.6. MSD Cytokine/Chemokine Analysis

Cytokines and chemokines from monocytes and fibroblasts were analyzed using the Meso Scale Discovery MSD^®^ U-PLEX development pack (Meso Scale Discovery, Rockville, MD, USA) with spots for IL-1, IL-6, IL-10, IL-8, VEGF, MIP-1a, MCP-1, and TNF-a according to the manufacturer’s protocol. Briefly, antibody solutions were created by diluting each biotinylated antibody to 10 µg mL^−1^, then added to 300 µL of the assigned linker, vortexed, and incubated at room temperature for 30 min. Then, 200 µL of stop solution was added, followed by vortexing and incubation for another 30 min; antibodies were then pooled. Coating solution was added at 50 µL per well, sealed, and incubated at room temperature for 1 h, followed by washing 3 times with PBS-T. To each well, 50 µL of sample, calibrator, or control was added and incubated for 1 h followed by washing. Detection antibody was then added at 50 µL per well, incubated for 1 h, washed, and read on the MESO QuickPlex SQ 120 (Meso Scale Discovery, Rockville, MD, USA).

#### 2.4.7. Immunofluorescence Microscopy

After washing cells with PBS to remove non-attached cells, samples were fixed at room temperature for 15 min with 4% paraformaldehyde. Cells were then permeabilized and blocked with 5% BSA and 0.1% TritonX-100 for 1 h at room temperature. Smooth alpha muscle antibody (a-SMA, Abcam, Cambridge, UK, ab5694) was diluted 1:200 in 2% BSA and incubated for 1 h at room temperature. After washing the samples, secondary antibodies (anti-rabbit, 488) and Phalloidin (647, Abcam, Cambridge, UK, ab176759) were added in 2% BSA at 1:200 dilution and incubated at room temperature for 1 h. Finally, samples were stained for nuclei using Hoechst diluted in DPBS at a 1:1000. Samples were imaged using the Zeiss Axiovert 40 C-FL ready 1 microscope (Zeiss, Oberkochen (Germany), and three representative images for each condition were taken at 60× and analyzed using Imaris x64.9.7.2 (Imaris, Zurich, Switzerland) with the threshold recommended for each channel.

### 2.5. Statistics

Results were expressed as mean values ± standard deviation (SD), with differences as determined by ANOVA for analysis of variance with Dunnett’s multiple comparison test or Mann–Whitney U tests. The results were considered significant when displaying *p*-values < 0.05.

## 3. Results

### 3.1. Fabrication of a Biocompatible Antifouling and Antimicrobial Sensor Coating

**Figure 1:**
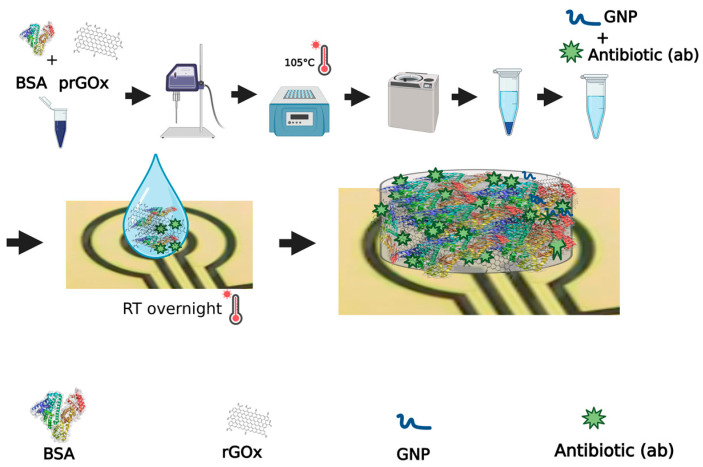
Schematic of BSA/prGOx/GNP/G nanocomposite synthesis showing ultrasonication of purified BSA and prGOx, BSA heat-denaturation, centrifugation and supernatant collection, addition of antibiotic and cross-linker to supernatant, drop-casting onto a plasma-treated gold electrode, and overnight incubation in a humidity chamber at room temperature (RT).

Biosensor surface modification approaches, such as antimicrobial coatings, are desirable because they could prevent initial bacterial adhesion and biofilm formation, enabling prolonged sensor application and wear time. To create a biocompatible coating for implantable biosensors that both prevents biofouling and exhibits enhanced antimicrobial protection, we set out to leverage the BSA/prGOx/GTA nanocomposite coating that we developed, which exhibits outstanding antifouling properties ex vivo [24]. However, to make the sensor implantable, we first replaced GTA with a more biocompatible crosslinker (Figure 1). This was accomplished by replacing GTA with GNP, which is a natural crosslinking agent with multiple advantages, including biocompatibility, non-cytotoxicity, and stability, positioning it as an eco-friendly and cost-effective alternative to traditional crosslinkers [28,33].

A nanocomposite’s biocompatibility is pivotal for the application of implantable or wearable biosensors and their prolonged use [30,31]. To assess the BSA/prGOx/GNP coating’s biocompatibility, primary human monocytes isolated from peripheral blood mononuclear cells (PBMCs) isolated from three donors were cultured on nanocomposite-coated wells and incubated at 37 °C. Cell viability was determined at various times using flow cytometry, and metabolic activity was measured using an alamarBlue™ Cell Viability Assay with periodic fluorescence intensity recordings. These studies revealed that the samples with the GTA-crosslinked BSA/prGOx/GTA coating displayed significantly reduced cell viability, akin to the cytotoxic control (PU film containing ZDEC), while exposure to BSA/prGOx/GNP did not significantly alter cell number or viability. These results confirm that replacing GTA with GNP as a crosslinker in BSA/prGOx/GNP is an effective way to reduce cell toxicity and that the resulting composite is more biocompatible than the original BSA/prGOx/GTA coating.

We then tested the coating’s electrochemical performance, which is key to achieving the sensor stability, longevity, and functionality required for long-term biosensing applications [15]. The coating’s electrochemical performance and antifouling properties were analyzed by using cyclic voltammetry (CV) and examining oxidation/reduction peaks and peak-to-peak distance (ΔEp) using potassium ferri/ferrocyanide. These studies revealed high current densities with oxidation/reduction peaks of 3.18/−2.53 × 10^−6^ A after coating the electrode with BSA/prGOx/GNP, compared to the peaks of an uncoated gold electrode (3.88/−3.00 × 10^−6^ A, respectively) (Supplementary Figure S1). The antifouling capacity was then confirmed by exposing the electrodes to a high concentration of a soluble protein (1% BSA), a typical blocking agent, revealing current densities of 2.02/−1.61 × 10^−6^ A of the BSA/prGOx/GNP coatings, whereas the gold electrode was immediately passivated and conductivity lost after exposure to this soluble protein.

### 3.2. Antimicrobial Efficacy and Stability Maintained over Time

**Figure 2:**
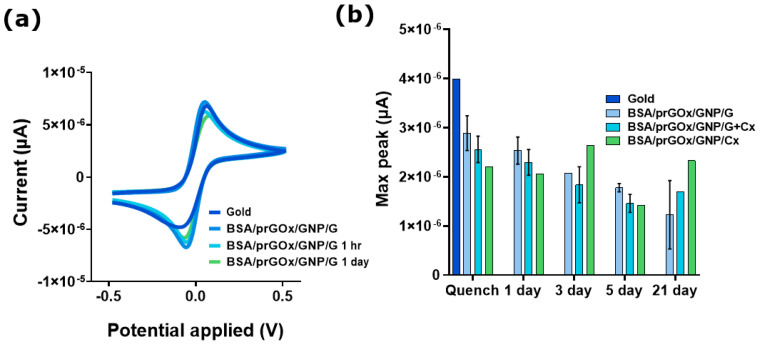
(**a**) BSA/prGOx/GNP/G coating current densities measured by cyclic voltammetry (CV). (**b**) Current density remained high for all BSA/prGOx/GNP/Ab coatings even after 21 days of incubation with 1% BSA; no signal was detected on the gold uncoated electrode (*n* = 2). The data are presented as mean ± standard deviation (SD).

To also instill this nanocomposite coating with antimicrobial protection, we leveraged the biocompatible cross-linker to covalently link the coating to antibiotics containing primary amine groups, such as gentamicin (G) and ceftriaxone (Cx) and one combining them, resulting in the creation of what we collectively termed BSA/prGOx/GNP/ab, or specifically BSA/prGOx/GNP/G, BSA/prGOx/GNP/Cx, and BSA/prGOx/GNP/G + Cx, respectively. G is an aminoglycoside broad-spectrum antibacterial that works against common implant-associated pathogens (e.g., *Staphylococcus* spp.) and aerobic Gram-negative organisms as well as established biofilms, while Cx is a third-generation cephalosporin used to treat a wide spectrum of infections [34,35]. Retention of good electrochemical properties was then verified by detecting high current densities with oxidation/reduction peaks of 7.31/−7.22 × 10^−6^ A after coating the electrode with BSA/prGOx/GNP/G, compared to the peaks of an uncoated gold electrode (6.79/−5.01 × 10^−6^ A, respectively) (Figure 2a). The antifouling capacity of each nanocomposite was then confirmed by exposing the electrodes to 1% BSA. High oxidation peaks of 6.48 × 10^−6^ A and 5.74 × 10^−6^ A and reduction peaks of −6.71 × 10^−6^ A and −6.17 × 10^−6^ A were seen for BSA/prGOx/GNP/G after exposure to 1% BSA for 1 h and 1 day, respectively, whereas the uncoated gold electrode was passivated immediately. Excellent transfer kinetics facilitating electron flow while mitigating protein diffusion were further displayed by a ΔEp of 0.091 V, 0.091 V, and 0.1309 V for BSA/prGOx/GNP/G immediately after coating as well as 1 h and 1 day in BSA, which were below or on par with 0.1309 V for the uncoated gold electrode (Figure 2a). Importantly, all BSA/prGOx/GNP/ab coatings, including BSA/prGOx/GNP/Cx and BSA/prGOx/GNP/G + Cx, maintained remarkably high current densities and low ∆Eps even after 3 weeks in 1% BSA (Figure 2b). These findings demonstrate the ability of BSA/prGOx/GNP/ab coatings to maintain high signal strength and assay sensitivity with little evidence of sensor passivation, further confirming its utility for long-term sensing.

**Figure 3:**
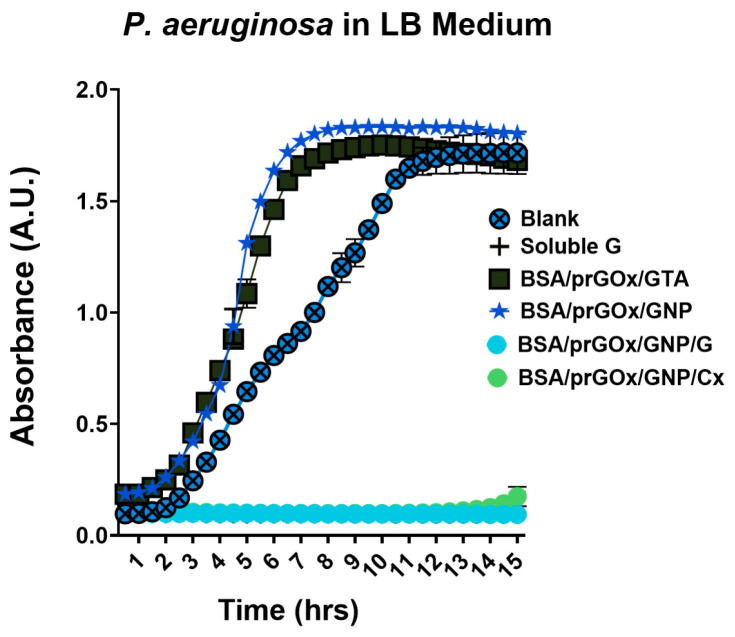
Evaluation of antimicrobial efficacy of nanocomposite coatings by a micro broth assay using ~10^6^ CFU/mL *P. aeruginosa* grown in LB medium over 15 h at 37 °C, measured at 450 nm. A saturating dose of soluble gentamicin (100 µg/mL) was used as a positive control (soluble G), and a blank tissue culture plate was used as a negative control (blank) (*n* = 3). The data are presented as mean ± standard deviation (SD). Note, some error bars are smaller than the symbols.

In addition, BSA/prGOx/GNP/G and BSA/prGOx/GNP/Cx both exhibited combined antifouling and antimicrobial properties, including excellent antimicrobial effectiveness against *P. aeruginosa*, a Gram-negative bacterium, when analyzed over 15 h in vitro (Figure 3). *P. aeruginosa* was chosen as a model organism for this study due to its well-documented persistence in medical devices, which is due to its strong adhesive nature, as well as its ability to form robust biofilms (i.e., structured communities of bacteria embedded in a self-produced fibrillar matrix [4,36]. Inhibition of bacterial cell growth and adhesion was on par with a saturating dose of soluble G (100 µg mL^−1^) and much less effective than the use of BSA/prGOx/GNP lacking an antimicrobial component or the original BSA/prGOx/GTA coating, as verified using a simple broth microdilution assay. To further enhance antimicrobial activity, we optimized the ratio of antibiotic to cross-linker (GNP) using BSA/prGOx/GNP/G as a model, testing combinations of two-fold dilutions from 1 mg mL^−1^ to 0.008 mg mL^−1^ of both G and GNP. The combination of G and GNP, each at 1 mg mL^−1^ (1:1 ratio), was found to exhibit optimal antimicrobial properties (Supplementary Figure S2). These results demonstrate that immobilization of the antibiotics on the nanocomposite through covalent cross-linking did not interfere with their antimicrobial activity.

To confirm that the BSA/prGOx/GNP/ab coatings exhibited sustained antimicrobial activity due to covalent crosslinking between the ε-amines of lysine residues in BSA and primary amine groups of the integrated antibiotics, we assessed the antimicrobial efficacy of BSA/prGOx/GNP/ab when fortified with meropenem (M), an antibiotic lacking primary amine or carboxyl functionalities. To compare this BSA/prGOx/GNP/M with BSA/prGOx/GNP/G and the original BSA/prGOx/GTA coatings, coated chips were inoculated with ~10^2^ CFU mL^−1^ of *P. aeruginosa*, incubated for 24 h at 37 °C, washed, and counted, with the process repeated daily for 3 days. Our hypothesis is supported by the finding that the amine-enriched BSA/prGOx/GNP/G reduced bacterial load by more than 100,000-fold and demonstrated superior antimicrobial potency compared to both BSA/prGOx/GTA and BSA/prGOx/GNP/M. Thus, attachment of the antibiotic to the BSA/prGOx/GNP/G backbone via covalent cross-linking proved crucial for sustained efficacy and minimal antibiotic leaching, which we considered a prerequisite for subsequent iterations.

**Figure 4:**
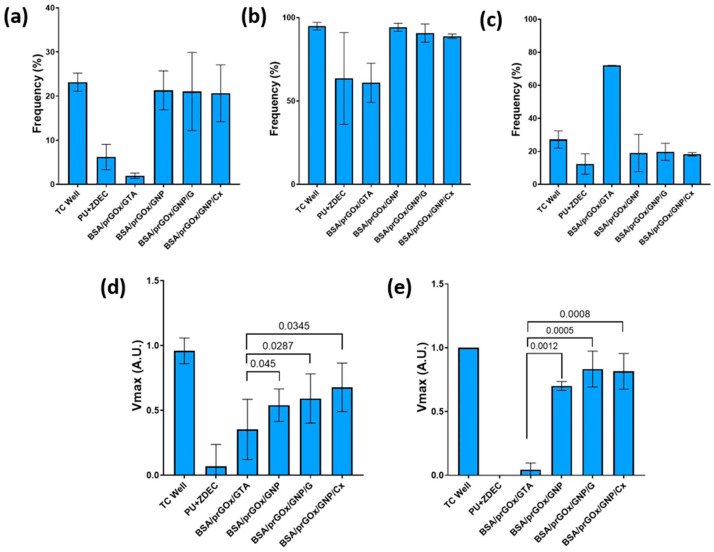
Monocytes seeded on BSA/prGOx/GTA, BSA/prGOx/GNP, BSA/prGOx/GNP/G, and BSA/prGOx/GNP/Cx, (**a**) Expression of the co-stimulatory molecule CD14 on live cells, (**b**) HLA-DR on CD14+ cells, and (**c**) CD83 on CD14+ cells, analyzed by flow cytometry (*n* = 2), followed by an alamarBlue cell viability assay after incubation for 24 h (*n* = 6) (**d**) and 48 h (*n* = 6) (**e**) with background signal removed before plotting data normalized. The biocompatible control was an empty plasma-treated polystyrene tissue culture plate well (TC Well), and the cytotoxic control was a polyurethane (PU) film containing 0.1% zinc diethyldithiocarbamate (ZDEC). The data are presented as mean ± standard deviation (SD).

To ensure that the biocompatibility of the coatings was maintained despite the incorporation of antibiotics, freshly seeded human-derived monocytes were seeded on the various nanocomposite coatings, a cytotoxic control PU film with ZDEC, and a biocompatible control (tissue culture plate). These immune cells also retained their functionality despite their contact with antibiotic-loaded BSA/prGOx/GNP/ab coatings (BSA/prGOx/GNP/G, BSA/prGOx/GNP/Cx) as indicated by continued expression of co-stimulatory molecules CD14, human leukocyte antigen DR (HLA-DR), and CD83 (Figure 4a–c). CD14 pertains to the classical CD14^++^CD16^−^ monocyte subpopulation, whereas CD14^−^CD16^+^ monocytes are precursors for pro-fibrotic M2 macrophages, which are crucial players in infection, inflammation, and disease pathogenesis [37,38,39]. Similarly, while indicative of immune deficiency in critically ill patients, HLA-DR also correlates with reduced antigen presentation and pro-inflammatory cytokine release, providing a prediction of mortality or risk of secondary infection [39,40]. Importantly, overall CD14 expression and subsequent HLA-DR expression on CD14^+^ cells were significantly higher in cells in contact with the BSA/prGOx/GNP/ab coatings, which appeared similar to the negative control, while their levels decreased dramatically on the BSA/prGOx/GTA coating and in the cytotoxic control film (Figure 4d,e). Additionally, the BSA/prGOx/GTA coating condition showed elevated CD83, an antigen-presenting cell activation marker, suggesting heightened monocyte activation, while this was not observed in cells on the BSA/prGOx/GNP/ab coatings [41,42]. Moreover, this improved biocompatibility was maintained despite the presence of crosslinked antibiotics in the coating, as verified in studies in which freshly isolated monocytes were cultured directly on BSA/prGOx/GNP/G and BSA/prGOx/GNP/Cx coatings (Figure 4d,e).

**Figure 5:**
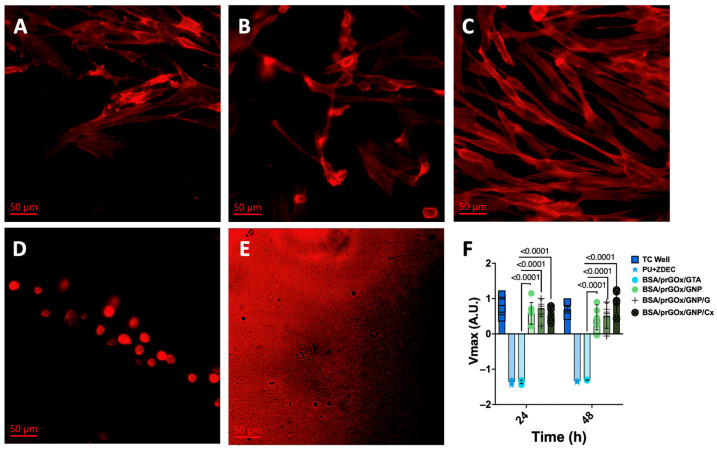
Immunofluorescent staining of fibroblasts seeded on (**A**) BSA/prGOx/GNP, (**B**) BSA/prGOx/GNP/G, (**C**) a plasma-treated tissue culture plate well (TC Well), (**D**) BSA/prGOx/GTA, and (**E**) the cytotoxic control (PU film with ZDEC) (*n* = 3). Images are showing ATTO 647 stained for F-actin. (**F**) alamarBlue viability assay of human-derived fibroblasts immediately after incubation with the nanocomposite coatings (BSA/prGOx/GNP, BSA/prGOx/GNP/G, BSA/prGOx/GNP/Cx, BSA/prGOx/GTA) at 37 °C for 24 h and 48 h (*n* = 3). The negative control was a plasma-treated tissue culture plate well (TC Well), and the positive control was the PU + ZDEC film. The background signal was removed before plotting, and the data were normalized. The data are presented as mean ± standard deviation (SD).

Given the integral role of stromal fibroblasts in tissue regeneration and wound recovery, as well as in the FBR, regulating fibroblast recruitment and adherence is crucial to improving the longevity and biocompatibility of implantable biosensors [7]. To explore this, primary human fibroblasts from three donors were cultured on the different nanocomposite coatings (BSA/prGOx/GNP, BSA/prGOx/GNP/G, BSA/prGOx/GNP/Cx, and BSA/prGOx/GTA), and both adhesion and viability were analyzed over time and compared to fibroblasts seeded on the biocompatible and cytotoxic control coatings. Neither BSA/prGOx/GNP/G, BSA/prGOx/GNP/Cx, nor unmodified BSA/prGOx/GNP (without antibiotics) exhibited any toxic effects on the cultured fibroblasts as the cell counts were similar to those measured in the biocompatible control wells without any coating (Figure 5). However, BSA/prGOx/GNP/G had fewer cells (*p* < 0.05), while BSA/prGOx/GTA and the cytotoxic control had significantly lower counts compared to the negative control (*p* < 0.001) (Supplementary Figure S3).

Fibroblast extension, a pivotal marker for strength of adhesion, provides insights into biocompatibility and the ensuing immunological response [6,41]. Fluorescence microscopic analysis of fibroblasts adherent to BSA/prGOx/GNP and BSA/prGOx/GNP/ab coatings (Figure 5A,B) revealed that fibroblasts spread to a similar moderate degree on both substrates. In contrast, the same cells spread to a much greater degree on standard tissue culture plastic substrates, which are especially treated to promote high levels of cell growth and matrix accumulation (Figure 5C), while the BSA/prGOx/GTA coating-treated cells were fewer in number, and those that remained adherent appeared unable to spread (Figure 5D), and the cytotoxic control (Figure 5E) showed no viable cells [6]. Importantly, the antifouling effect did not compromise cell metabolic activity, as verified by the alamarBlue assay conducted immediately after incubation at 37 °C for 60 min, with the background signal removed. Once again, the assay demonstrated increased metabolic activity (*p* < 0.0001) in fibroblasts exposed to BSA/prGOx/GNP, BSA/prGOx/GNP/G, and BSA/prGOx/GNP/Cx compared to BSA/prGOx/GTA coating conditions for both 24 and 48 h incubation periods (Figure 5F).

### 3.3. Cytokine Production

**Figure 6:**
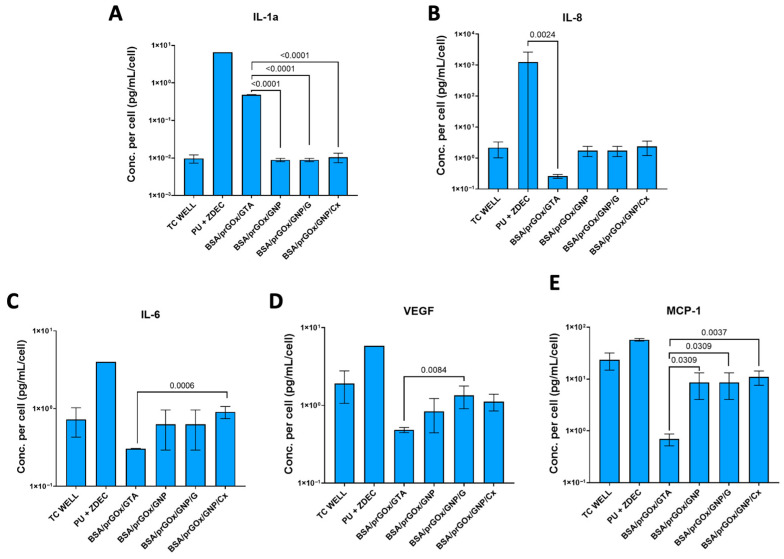
Fibroblast cytokine expression of IL-1α (**A**), IL-8 (**B**), IL-6 (**C**), VEGF (**D**), and MCP-1 (**E**) after 24 h incubation. The biocompatible control was a plasma-treated tissue culture plate well (TC Well), and the cytotoxic control was PU + ZDEC. The data were normalized by cell count. Expression was upregulated after exposure to BSA/prGOx/GTA for IL-1α and was not significant for BSA/prGOx/GNP/Ab coatings except when compared to BSA/prGOx/GNP/Cx for IL-6, VEGF, and MCP-1. The data are presented as mean ± standard deviation (SD). Significance was calculated by ANOVA followed by Dunnett’s multiple comparisons. Note some error bars are smaller than the symbols.

Monitoring pro-inflammatory cytokine expression is essential for optimal biomaterial design and functionality, as the release of high levels of cytokines, such as IL-6 and TNF-α, suggests an enhanced inflammatory response and potential material rejection, while anti-inflammatory cytokines like IL-10 indicate better material integration [43,44]. To discern the impact of BSA/prGOx/GNP/ab on these responses, we analyzed the expression of key pro- and anti-inflammatory markers (IL-6, IL-1α, IL-10, IL-8, VEGF, MIP-1α, MCP-1, and TNF-α) in fibroblast supernatants after 1 day of contact with the coating, and results were normalized to cell counts [43,44]. For BSA/prGOx/GNP, the levels of IL-1α, IL-8, IL-6, VEGF, and MCP-1 closely mirrored the biocompatible control plastic dish, suggesting a neutral cellular response, which is often preferable for implanted devices (Figure 6) [4,43]. MIP-1α, IL-10, and TNF-α secretion were either absent or under the limit of detection (LoD) for all conditions. Compared to fibroblasts exposed to BSA/prGOx/GNP, cells exposed to the BSA/prGOx/GTA coating upregulated IL-1α expression (*p* < 0.0001) and significantly downregulated IL-6 (*p* < 0.001) and MCP-1 (*p* < 0.01) when compared to BSA/prGOx/GNP/Cx and VEGF (*p* < 0.01) compared to BSA/prGOx/GNP/. BSA/prGOx/GNP exhibited marked suppression of pro-inflammatory IL-1α and overall cytokine levels compared to the negative control, again underscoring its non-inflammatory nature. However, the slight cytokine elevation observed in cells on BSA/prGOx/GNP/Cx could suggest a reduced level of biocompatibility for Cx when compared to G as an antimicrobial component.

### 3.4. Functionality as Immunoassay

For further validation, we leveraged the exceptional electrochemical properties of BSA/prGOx/GNP/G to develop an immunosensor that uses a sandwich ELISA detection method wherein the working electrodes are selectively functionalized with capture antibodies (cAbs) directed against different cytokines (MIP-1β and IL-6) using EDC/NHS chemistry. The cAb-immobilized immunosensor was exposed to different concentrations of the target antigens, and detection was achieved using biotinylated secondary antibodies based on the reduction in poly-streptavidin–horseradish peroxidase (HRP) binding followed by tetramethylbenzidine (TMB) precipitation.

**Figure 7:**
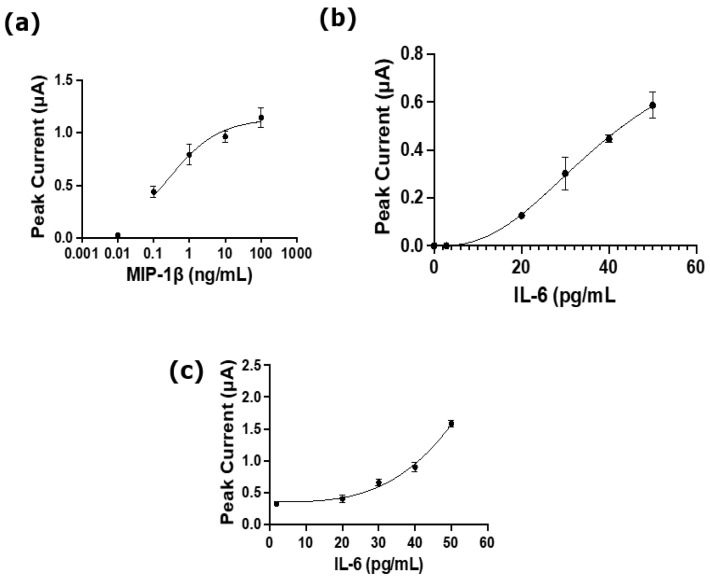
(**a**) The immunosensor developed with BSA/prGOx/GNP and functionalized with capture antibodies (cAb) to detect MIP-1b showed a response in the range of 0.10 to 100 ng mL^−1^ in RPMI medium (*n* = 3); (**b**) the immunosensor with BSA/prGOx/GNP/G functionalized with capture antibodies (cAb) to detect IL-6 in RPMI medium showed a response in the range of 20 to 50 pg mL^−1^ (*n* = 3); and (**c**) the immunosensor developed with BSA/prGOx/GNP and functionalized with cAbs to detect IL-6 showed a limit of detection of 0.018 ng/mL in human plasma (*n* = 3), indicating its high sensitivity even in complex biological samples. The data are presented as mean ± standard deviation (SD).

First, we explored the potential of the BSA/prGOx/GNP/G-coated immunosensor to detect MIP-1β and IL-6 in RPMI culture medium. The immunosensor response was calibrated against different concentrations of MIP-1β and IL-6 in the range of 0.10 to 100 ng mL^−1^ and 20 to 50 pg mL^−1^, respectively (Figure 7a,b). LoDs of 0.096 ng mL^−1^ and 0.028 ng mL^−1^ were obtained for MIP-1β and IL-6, respectively, in this culture medium. To evaluate immunosensing performance in more complex biological samples, we calibrated a BSA/prGOx/GNP/G-coated immunosensor using spiked concentrations of IL-6 in human plasma (Figure 7c) and obtained a LoD of 0.018 ng mL^−1^. This is on par with the previously published performance of the BSA/prGOx/GTA-coated immunosensor, which obtained a LoD of 0.013 ng mL^−1^ [27]. These results confirm the unique ability of BSA/prGOx/GNP/G to maintain high sensor sensitivity even in complex biological fluids.

## 4. Discussion

The rise in personalized medicine and digital health necessitates the development of stable implantable/wearable biosensors for continuous, long-term monitoring of analytes, such as glucose, lactate, or cytokines, to enable real-time health management. Compared with traditional implanted devices (e.g., pacemakers, heart stents), which are designed to function for years inside the body, biosensors face considerable challenges in maintaining performance over extended times due to multiple failures in the complex, dynamic in vivo environment [45,46,47]. These challenges include biofouling, immunogenic responses, FBR, material degradation, as well as interference from complex physiological fluids [48]. Such issues contribute to shorter functional lifetimes and reduced accuracy over time [2]. Consequently, enhancing the biocompatibility, antifouling properties, and overall stability of biosensor coatings remains crucial to meet the growing demands for reliable, long-term monitoring solutions in clinical and personalized health applications.

### 4.1. Main Findings and Contributions

Addressing this, we successfully synthesized a new prototype using a cost-effective antimicrobial, antifouling, and biocompatible sensor coating formulation. To develop a biocompatible coating for implantable biosensors with antifouling and antimicrobial properties, the original BSA/prGOx/GTA nanocomposite was modified by replacing GTA with GNP, a natural, biocompatible, and eco-friendly crosslinker [29,49].

Biocompatibility testing using primary human monocytes and fibroblasts demonstrated significantly reduced cytotoxicity with the BSA/prGOx/GNP coating compared to the original GTA-based formulation, which displayed toxicity similar to that exhibited by a known cytotoxic control. These findings highlight GNP’s superiority as a crosslinker for enhancing the safety and applicability of biosensor coatings. To enhance antimicrobial protection, the nanocomposite coating was covalently linked to antibiotics (gentamicin, ceftriaxone, or both) using GNP, resulting in BSA/prGOx/GNP/ab variants. These coatings demonstrated strong antifouling and antimicrobial properties, effectively inhibiting microbial growth and adhesion comparable to a saturating dose of soluble antibiotics, confirming that covalent immobilization of antibiotics on the nanocomposite preserves their antimicrobial efficacy. Finally, these coatings exhibited excellent electrochemical properties, antifouling capacity, and stable performance over 3 weeks in 1% BSA, maintaining high current densities, low ΔEp values, and robust assay sensitivity, demonstrating their suitability for long-term biosensing applications.

Thus, this coating dually combats contamination by deterring microbial adhesion and neutralizing nearby microbial activity while remaining safe to mammalian cells. The adaptability and versatility of the BSA/prGOx/GNP/ab coating make it suitable for a wide range of sensing needs in the biomedical and industrial fields, such as next-generation wearables, implantable biosensors, in vitro diagnostics, and bioreactors. Furthermore, its ability to maintain high sensor sensitivity and stability over extended periods (3 weeks or more) highlights its potential to enhance long-term performance in complex biological environments. While the intricate and gradual onset of FBR and biofilm formation in vivo limits direct translation of our in vitro data, these initial findings underline the immense potential of BSA/prGOx/GNP/ab to revolutionize the biocompatibility, durability, and functionality of both wearable and implantable biosensors.

### 4.2. Future Research

Nevertheless, comprehensive testing and validation are imperative to mature this technology, including demonstration of clinical efficacy and carrying out safety evaluations. While *P. aerigunosa*’s adhesive properties and biofilm-forming capabilities make it an ideal candidate for studying microbial colonization and infection control strategies in healthcare environments, future studies should include testing of other common infection-inducing microbes such as *Staphyloccocus aureus*, *Escherichia coli*, and *Candida albicans* [36,50,51,52]. Gauging the antimicrobial effect of BSA/prGOx/GNP/ab against a wide range of microbes under physiologically relevant conditions such as flow and the presence of human blood is crucially important. Such evaluations will elucidate the combined impact of its antimicrobial and antifouling properties. Furthermore, assessing its biocompatibility through evaluations such as thrombogenicity and long-term stability is vital to ensure its effectiveness in vivo. Additionally, several sensor-related queries that still require in-depth electrochemical analysis using methods such as chronoamperometry, single-frequency electrochemical impedance spectroscopy, in-line sensing tests, and clinical accuracy employing clinical error grid analysis in vitro are imperative to advance these technologies toward in vivo evaluations.

## 5. Patents

S.W.-M., P.J., and D.E.I. are listed as inventors on a patent describing this technology.

## Data Availability

The data supporting these findings are available upon reasonable request from the corresponding author.

## Supplementary Materials

The following supporting information can be downloaded at https://www.mdpi.com/article/10.3390/bios15030171/s1. Figure S1: BSA/prGOx/GNP coating current densities measured by cyclic voltammetry (CV) compared to uncoated control (gold) and after 1 h incubation with BSA (n = 2). Figure S2: Antimicrobial efficacy of BSA/prGOx/GNP/G coatings with varied antibiotic (G) (a,b) and crosslinker (GNP) (c,d) concentrations measured by a micro broth assay using 10^6^ CFU/mL *P. aeruginosa* grown in LB medium over 15 h at 37 °C (a,c), then washed and grown again with fresh bacteria for 15 h (b,d) (n = 3). A saturating dose of soluble gentamicin (100 µg mL^−1^) was used as a positive control (Soluble G), and a blank tissue culture plate was used as a negative control (TC Well). Figure S3: AlamarBlue assay run immediately on human-derived fibroblasts after 24 h and 48 h incubation with BSA/prGOx/GNP/Ab coatings (BSA/prGOx/GNP, BSA/prGOx/GNP/G, BSA/prGOx/GNP/Cx) and BSA/prGOx/GTA at 37 °C for 24h (a–c) and 48 h (d–f); background signal was removed before plotting. The negative control was a plasma-treated tissue culture plate well (TC Well), and the positive control was polyurethane (PU) film containing 0.1% zinc diethyldithiocarbamate (ZDEC) (PU+ZDEC).

## Abbreviations

The following abbreviations are used in this manuscript:

ab	antibiotics
BSA	bovine serum albumin
C	cefepime
cAb	capture antibodies
Co	colistin
Cx	ceftriaxone
CV	cyclic voltammograms
DI	deionized
FBR	foreign body response
G	genamicin
GNP	genipin
GTA	glutaraldehyde
HLA-DR	human leukocyte antigen DR
HRP	horseradish peroxidase
LoD	limit of detection
M	meropenum
prGOx	pentamine functionalized reduced graphene oxide flakes
PU	polyurethane
TMB	tetramethybenzidine
ZDEC	zinc diethyldithiocarbamate
