# Supplementary material for: An Antimicrobial and Antifibrotic Coating for Implantable Biosensors
Source: Biosensors (Basel). 2025 Mar 6;15(3):171. doi: 10.3390/bios15030171 (PMC11940466; doi:10.3390/bios15030171)
Supplement: Supplementary file 1 [file biosensors-15-00171-s001.zip › biosensors-3457099-supplementary.pdf]

biosensors

MDPI

Supplementary

1.1 Fabrication of gold electrode chips

The gold electrode chips consist of 6 planar electrodes - 4 working electrodes, a common counter and reference electrode, all fabricated on glass substrates with a form factor of ~1x2cm. The cell for electrochemical measurements is created by placing a sample well beneath a custom laser-cut double-sided adhesive tape, and it only requires about 15 µL to cover all the electrodes. The individual working electrodes can be separately functionalized with specific capture probe enabling multiplexed detection using a sample volume as low as 15 ul. The reproducibility of coatings has been previously measured using the antifouling composition (BSA/prGOx/GTA) as described in our past publications.

1.2 Antimicrobial efficacy and leaching

To assess potential antibiotic leaching from the nanocomposite, we assessed chips with three nanocomposite coatings: 1.) without antibiotics (prGOx/BSA/GNP), 2.) with antibiotics containing a primary amine-containing functional group (prGOx/BSA/GNP/G), and 3.) an antibiotic with no primary amine groups (meropenum, prGOx/BSA/GNP/M). Initially, two pieces of double-sided tape were placed to create a well in which LB medium and bacterial suspension could remain in contact with the nanocomposite coating. Then 40 µL of ~ 10^2 CFU /mL *P. aeruginosa* in LB medium was added to the nanocomposite and incubated in a humidity chamber for 24 h on a shaker at 70 rpm and 37 °C. The following day, the remaining inoculum was collected and the nanocomposite was washed with 60 µL LB medium, serially diluted, plated, and counted after 24 h incubation at 37 °C. This procedure was then repeated daily for two consecutive days. Collected media was added to a 5mm piece of ø filter paper (Thermo Fisher Scientific) placed on an LB agar plate which had been swabbed with *P. aeruginosa* in LB medium to create a bacterial lawn. As a control, a separate plate was swabbed and 1 µL of 70 mg mL-1 G was diluted at 1:20 in LB medium, 2-fold serially diluted, and added to 5 mm pieces of ø filter paper on the bacterial lawn. Plates were then incubated for 24 h at 37 °C whereafter the zones of inhibition around the filter papers were analyzed.

 https://doi.org/10.3390/10.3390/bios15030171

Figures

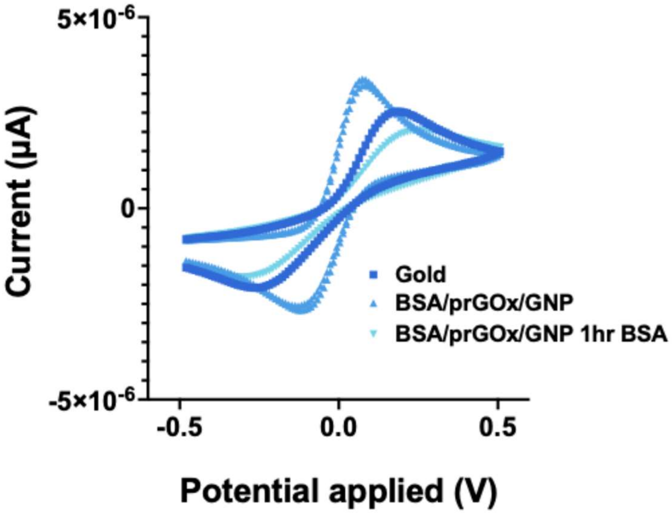

Figure S1. BSA/prGOx/GNP coating current densities measured by cyclic voltammetry (CV) compared to uncoated control (gold) and after 1 hr incubation with BSA (n=2).

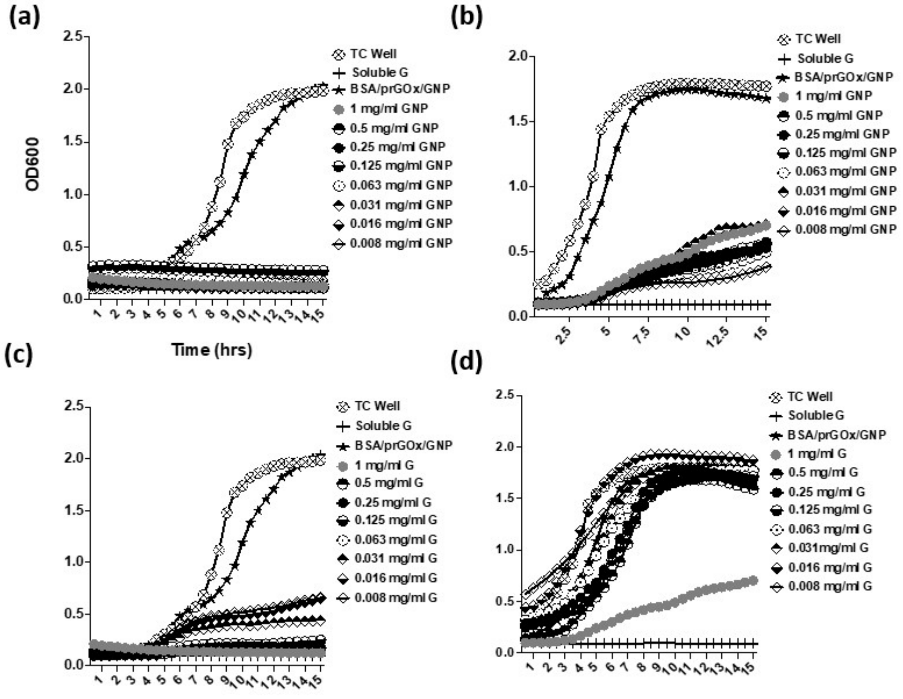

Figure S2. Antimicrobial efficacy of BSA/prGOx/GNP/G coatings with varied antibiotic (G) (a and b) and crosslinker (GNP) (c and d) concentrations measured by a micro broth assay using 10^6 CFU/mL *P. aeruginosa* grown in LB medium over 15 h at 37 °C (a and c) , then washed and grown again with fresh bacteria for 15 h (b and d) (n=3). A saturating dose of soluble gentamicin (100 µg mL^{-1}) was used as a positive control (Soluble G), and a blank tissue culture plate was used as a negative control (TC Well).

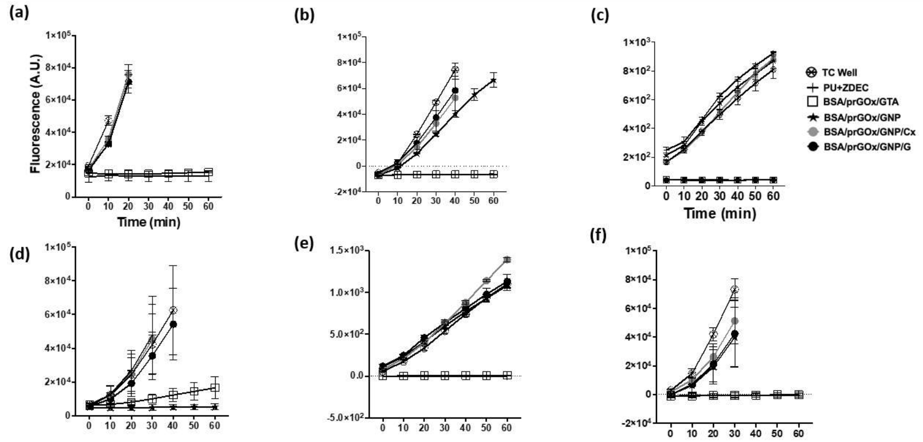

Figure S3. AlamarBlue assay run immediately on human-derived fibroblasts after 24 h and 48 h incubation with BSA/prGOx/GNP/Ab coatings (BSA/prGOx/GNP, BSA/prGOx/GNP/G, BSA/prGOx/GNP/Cx) and BSA/prGOx/GTA at 37 °C for 24h (a, b, c) and 48 h (d, e, f); background signal was removed before plotting. The negative control was a plasma-treated tissue culture plate well (TC Well), and the positive control was polyurethane (PU) film containing 0.1% zinc diethyl-dithiocarbamate (ZDEC) (PU+ZDEC).
